# Polymorphism in the *MHC2TA* Gene Is Associated with Features of the Metabolic Syndrome and Cardiovascular Mortality

**DOI:** 10.1371/journal.pone.0000064

**Published:** 2006-12-20

**Authors:** Eero Lindholm, Olle Melander, Peter Almgren, Göran Berglund, Carl-David Agardh, Leif Groop, Marju Orho-Melander

**Affiliations:** Department of Clinical Sciences, Clinical Research Center, University Hospital Malmö (MAS), Lund University Malmö, Sweden; Institute for Genomic Research, United States of America

## Abstract

**Background:**

Recently, a -168A→G polymorphism in the MHC class II transactivator gene (MHC2TA) was shown to be associated with increased susceptibility to myocardial infarction (MI).

**Aim:**

To confirm the association between the MHC2TA -168A→G polymorphism and MI and to study its putative role for microalbuminuria, the metabolic syndrome (MetS) and cardiovascular mortality.

**Materials and Methods:**

Using an allelic discrimination method we genotyped 11,064 individuals from three study populations: 1) 4,432 individuals from the Botnia type 2 diabetes (T2D) study, 2) 1,222 patients with MI and 2,345 control subjects participating in the Malmö Diet and Cancer study and comprising an MI case-control sample, and 3) 3,065 T2D patients from the Local Swedish Diabetes registry.

**Results:**

No association between the -168A→G polymorphism in MHC2TA and MI was observed. However, in the Botnia cohort the AG/GG genotypes were associated with cardiovascular mortality after MI (1.78 [1.09–2.92], p = 0.02). In addition, the AG/GG genotypes were more common in subjects with MetS (40.1% vs. 36.9%, p = 0.03) and in non-diabetic subjects with microalbuminuria (45.4% vs. 36.5%, p = 0.003) compared to control subjects.

**Conclusions:**

A polymorphism in MHC2TA was associated with cardiovascular mortality and predictors of cardiovascular mortality, microalbuminuria and MetS.

## Introduction

Cardiovascular disease is the leading cause of death in Sweden and in most Western countries. Almost 50% of deceased individuals died due to cardiovascular causes in Sweden 2005 (Swedish National Board of Health and Welfare http://www.socialstyrelsen.se). Both genetic and environmental factors modify the risk for cardiovascular diseases including myocardial infarction (MI) [1],smoking, dyslipidaemia, diabetes, obesity and hypertension, are present in up to 90% of patients with MI [2], [3]. Family history of MI has been shown to be a strong independent risk factor for coronary heart disease [4]–[7].

Microalbuminuria is an established risk marker for cardiovascular morbidity and mortality both in diabetic [8] and non-diabetic patients [9]. Inflammation is believed to play a major role in the pathogenesis of both microalbuminuria [10] and MI [11]. Insulin resistance has been proposed as a common denominator for these conditions, and has also been related to subclinical chronic inflammation [12].

A –168 A→G polymorphism in the MHC class II transactivator gene (*MHC2TA*) was recently found to be associated with MI, rheumatoid arthritis and multiple sclerosis [13]. The -168 A→G polymorphism was associated with lower expression of *MHC2TA* after stimulation of leukocytes with interferon-γ in humans and differences in expression of MHC class II molecules in different rat strains. Because of the role of MHC class II molecules in recognition of antigen molecules, genes like *MHC2TA* that can influence expression of MHC class II, are also candidate genes for autoimmune diseases [13].

To address this issue, we searched for any association between *MHC2TA* -168 A→G polymorphism and cardiovascular morbidity and mortality as well as their predictors, microalbuminuria and the metabolic syndrome (MetS).

## Materials and Methods

### Study Subjects

Patients were selected from three large populations in Finland and Sweden; the Botnia study, the Malmö Diet and Cancer Study (MDC) and the Diabetes Registry in Southern Sweden (DR). The protocols were approved by local Ethics committees, and informed consent was obtained from all subjects.

#### The Botnia Study

The Botnia Study was initiated in 1990 and represents a large population-based type 2 diabetes (T2D) family study in Finland and Sweden, aiming at identification of genes increasing susceptibility to T2D, MetS and associated complications. Details of the study cohort, sampling strategy as well as anthropometric and metabolic measurements have been described earlier [14], [15]. At the baseline examination, a structured questionnaire was completed by specially trained nurses, covering information about diseases other than T2D (particularly hypertension, coronary heart disease, MI and stroke) and data on smoking habits. Both previous and current smokers were recorded as smokers. Diagnosis of MI was always established in the hospital. Microalbuminuria was defined as urinary albumin excretion rate >20 µg/min in an overnight urine collection.

Total and cardiovascular mortality were assessed with a median follow up time of 7.9 years and the mortality data was obtained from the central death-certificate registry in Finland. Cardiovascular mortality was classified using the 9th revision of the International Classification of Diseases (cardiovascular diagnosis codes 390–459) before 1997 and the 10th revision (codes 100–199) thereafter. Causes of death were classified as 1) cardiovascular death (coronary heart disease), cerebrovascular disease (including both thrombotic stroke and cerebral haemorrhage) or other cardiovascular (including pulmonary embolism, abdominal aortic aneurysm, hypertensive complications, general atherosclerosis and peripheral artery disease with gangrene) or 2) other causes of death (neoplasm, violent or other). MetS was defined according to the National Cholesterol Education Program (NCEP) [16].

In total, 4,432 individuals were genotyped for the *MHC2TA* -168 A→G polymorphism including 2,864 individuals without diabetes mellitus and 1,557 with T2D. Data on MI was available in 97% and on microalbuminuria in 64% of the subjects. Data on cardiovascular mortality was available for all patients.

#### The MI case- control population from the Malmö diet and cancer study (MDC)

The Malmö Diet and Cancer study population (MDC) [17] includes 28,098 randomly selected men (born 1923–1945) and women (born 1923–1950) living in the city of Malmö (population 250,000) in Sweden. A baseline examination was carried out between 1991 and 1996 encompassing a comprehensive assessment of lifestyle factors, heredity, medication as well as previous and current diseases. On December 31^st^, 2000 the study population was checked against the Swedish National Board of Health and Welfare's National Patient Registry and Cause of Death Registry. MI cases (first MI) were identified in the Swedish Patient Registry or in the Swedish Cause of Death Registry; using ICD 9–10 codes 410 and I21 in the Swedish Patient Registry and 410–414 and I21–I25 in the Swedish Cause of Death Registry.

Two age- (±1 year) and gender-matched controls without MI from MDC were assigned to each MI patient, resulting in a case-control material consisting of 1,244 MI patients and 2,488 controls. Of total 3,732 individuals, 3,657 were successfully genotyped for the A→G polymorphism in *MHC2TA*. Diabetes diagnosis was defined as self reported earlier diagnosis, fasting blood glucose ≥ 6.1 mmol/l and/or treatment for earlier diagnosed diabetes mellitus.

#### Diabetes registry in Southern Sweden (DR)

3,065 T2D patients from a local diabetes registry in Southern Sweden [18], most of them enrolled at the Department of Endocrinology, University Hospital MAS, Malmö, were selected and genotyped for the *MHC2TA* -168 A→G polymorphism. Data on MI was obtained from the patient records and was available in 77% and on microalbuminuria in 70% of the subjects.

#### Analytical techniques

HbA_1c_, total cholesterol, HDL-cholesterol, triglycerides and P-creatinine were measured with standard laboratory methods. A detailed description of the methods are found elsewhere [15], [18], [19]. The urinary albumin concentration was in DR determined first by immunonephelometric method (Beckman Instruments, CA, USA) until 1998, and thereafter by an immunoturbimetric method (Beckman Coulter, Beckman Instruments, CA, USA). In the Botnia study, urine albumin concentration was determined with a radioimmunoassay with a detection limit of 2 mg/l. Microalbuminuria was defined as AER of ≥20 µg/min.

#### Genotyping

In total 11,064 individuals were successfully genotyped for the -168 A/G polymorphism (rs3087456) using allelic discrimination method on the ABI 7900 instrument (Applied Biosystems, Foster City, CA). Risk genotypes were defined according to earlier published report [13]. The genotyping success rate was 97.9, 98.0 and 99.0% in Botnia, MDC and DR cohorts, respectively.

### Statistical methods

Data are presented as mean ± SD or as median [25^th^–75^th^] percentile. Chi-square tests were used to analyze differences between allele- and genotype frequencies. To test differences between group means, the Student's two-tailed t-test was used for normally distributed values and Mann-Whitney U-test for non-normally distributed medians. In order to identify factors associated with MI and microalbuminuria, a multiple logistic regression analysis with forward selection was performed. Because of the nature of Botnia cohort as a family collection, the analyses were adjusted for the family relations. For the mortality analyses, clinical variables together with *MHC2TA* genotypes were entered into a forward stepwise Cox regression model adjusted for sex, age and family relations.

All data were analyzed with a NCSS 2004 (NCSS statistical software, Kaysville, UT, USA). A p-value of <0.05 was considered statistically significant. Power analysis was made using Genetic Power Calculator [20].

## Results

The clinical characteristics of the study groups are given in Table 1. The genotype and allele frequencies of the *MHC2TA* polymorphism were similar in patients with or without MI, regardless of the study population and T2D status (Table 2). No association with T2D was observed, neither in the Finnish (Botnia), nor in the Swedish (MDC or DR) cohorts.

**Table 1:**
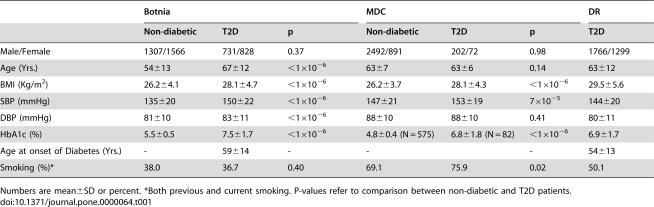
Clinical characteristics of the study subjects.

	Botnia			MDC			DR
	Non-diabetic	T2D	p	Non-diabetic	T2D	p	T2D
Male/Female	1307/1566	731/828	0.37	2492/891	202/72	0.98	1766/1299
Age (Yrs.)	54±13	67±12	<1×10^−6^	63±7	63±6	0.14	63±12
BMI (Kg/m^2^)	26.2±4.1	28.1±4.7	<1×10^−6^	26.2±3.7	28.1±4.3	<1×10^−6^	29.5±5.6
SBP (mmHg)	135±20	150±22	<1×10^−6^	147±21	153±19	7×10^−5^	144±20
DBP (mmHg)	81±10	83±11	<1×10^−6^	88±10	88±10	0.41	80±11
HbA1c (%)	5.5±0.5	7.5±1.7	<1×10^−6^	4.8±0.4 (N = 575)	6.8±1.8 (N = 82)	<1×10^−6^	6.9±1.7
Age at onset of Diabetes (Yrs.)	-	59±14	-	-		-	54±13
Smoking (%)*	38.0	36.7	0.40	69.1	75.9	0.02	50.1

Numbers are mean±SD or percent. *Both previous and current smoking. P-values refer to comparison between non-diabetic and T2D patients.

**Table 2:**
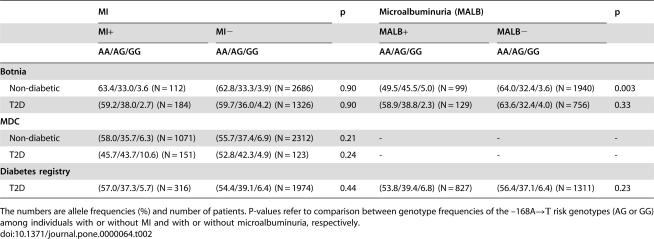
The genotype frequencies of the *MHC2TA* -168 A→G polymorphism in different study populations according to history of previous MI and microalbuminuria status.

	MI		p	Microalbuminuria (MALB)		p
	MI+	MI−		MALB+	MALB−	
	AA/AG/GG	AA/AG/GG		AA/AG/GG	AA/AG/GG	
**Botnia**						
Non-diabetic	63.4/33.0/3.6 (N = 112)	(62.8/33.3/3.9) (N = 2686)	0.90	(49.5/45.5/5.0) (N = 99)	(64.0/32.4/3.6) (N = 1940)	0.003
T2D	(59.2/38.0/2.7) (N = 184)	(59.7/36.0/4.2) (N = 1326)	0.90	(58.9/38.8/2.3) (N = 129)	(63.6/32.4/4.0) (N = 756)	0.33
**MDC**						
Non-diabetic	(58.0/35.7/6.3) (N = 1071)	(55.7/37.4/6.9) (N = 2312)	0.21	-	-	-
T2D	(45.7/43.7/10.6) (N = 151)	(52.8/42.3/4.9) (N = 123)	0.24	-	-	-
**Diabetes registry**						
T2D	(57.0/37.3/5.7) (N = 316)	(54.4/39.1/6.4) (N = 1974)	0.44	(53.8/39.4/6.8) (N = 827)	(56.4/37.1/6.4) (N = 1311)	0.23

The numbers are allele frequencies (%) and number of patients. P-values refer to comparison between genotype frequencies of the –168A→Τ risk genotypes (AG or GG) among individuals with or without MI and with or without microalbuminuria, respectively.

**Figure 1:**
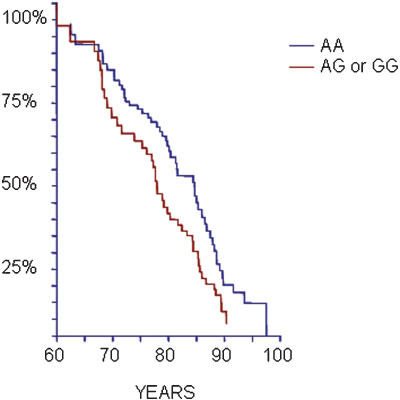
Cardiovascular mortality in the Botnia cohort in patients with previous MI according to MHC2TA -168 A→G genotypes. Kaplan Meier survival curves illustrating a higher risk for CV mortality (HR 1.76 [1.09–2.82], p = 0.02) in AG/GG genotype carriers with previous history of MI.

No correlation between the *MHC2TA* -168 AG/GG genotypes and cardiovascular mortality was found in the Botnia Study population (HR 0.96 [0.75–1.22], p = 0.74) (Table 3). As the *MHC2TA* polymorphism was earlier shown to be associated with MI, we performed a subgroup analysis of individuals with previous history of MI. In fact, among these patients the *MHC2TA* AG/GG genotypes were associated with increased risk of cardiovascular death compared with AA genotype carriers (HR 1.76 [1.09–2.82], p = 0.02) (Table 3, Figure 1). We also tested the *MHC2TA* GG genotype against the AA or AG genotypes and found that the GG genotype was protective against cardiovascular death in the whole group (HR 0.38 [0.16–0.92], p = 0.03), but not in patients with previous MI (HR 0.45 [0.49–4.16], p = 0.48).

**Table 3:**
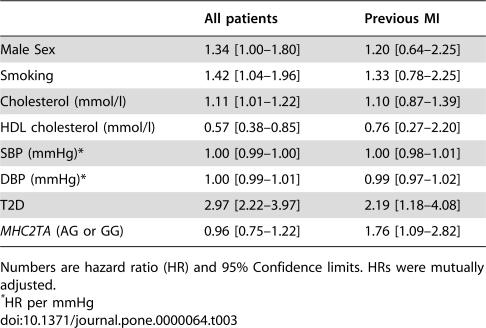
Predictors of cardiovascular mortality among all individuals and patients with previous MI from the Botnia study.

	All patients	Previous MI
Male Sex	1.34 [1.00–1.80]	1.20 [0.64–2.25]
Smoking	1.42 [1.04–1.96]	1.33 [0.78–2.25]
Cholesterol (mmol/l)	1.11 [1.01–1.22]	1.10 [0.87–1.39]
HDL cholesterol (mmol/l)	0.57 [0.38–0.85]	0.76 [0.27–2.20]
SBP (mmHg)*	1.00 [0.99–1.00]	1.00 [0.98–1.01]
DBP (mmHg)*	1.00 [0.99–1.01]	0.99 [0.97–1.02]
T2D	2.97 [2.22–3.97]	2.19 [1.18–4.08]
*MHC2TA* (AG or GG)	0.96 [0.75–1.22]	1.76 [1.09–2.82]

Numbers are hazard ratio (HR) and 95% Confidence limits. HRs were mutually adjusted.

* HR per mmHg

The *MHC2TA* AG/GG genotypes were more frequently found among patients with MetS (40.1 vs. 36.9%, p = 0.030) as well as among non-diabetic individuals with microalbuminuria in the Botnia cohort (50.0% vs. 36.0%, p = 0.003, Table 2). In contrast, the AG/GG genotypes were not associated with microalbuminuria among T2D patients, neither in the Botnia, nor the DR cohort (Table 2). Correspondingly, logistic regression analysis with age, waist-hip ratio, fasting insulin, systolic- and diastolic blood pressure, smoking, gender and *MHC2TA* AG/GG genotypes as independent factors, revealed the AG/GG genotypes as risk factors for microalbuminuria in non-diabetic subjects (OR 2.07 [1.35–3.18], p = 0.0009), but not in diabetic patients (OR 1.22 [0.80–1.84], p = 0.35).

The statistical power to detect differences in risk of MI according to genotype assuming dominant model and genotype relative risk of 1.2 was 32.0% in Botnia, 95.1% in the MDC cohort and 36.5% for Swedish T2D patients (from DR). The corresponding figures when assuming genotype relative risk of 1.5 were 95.0, 100.0, and 92.7%. In the pooled Swedish sample the power was 97.0% assuming a relative risk of 1.2 and 100% at the level of 1.5.

## Discussion

The key finding of the present study was an association between the *MHC2TA* -168 A→G polymorphism and cardiovascular mortality as well as with predictors thereof, microalbuminuria and MetS. These findings support the earlier report of association between this polymorphism and MI [13]. However, in contrast to the earlier study [13], the -168 A→G was not associated with MI in our study. One possible explanation could be differences in definition of MI. Our study consisted of population based material, where the information of MI was collected retrospectively, whereas the study population of Swanberg *et al*. was recruited from all patients below 60 yrs. that were admitted to the hospital for acute MI.

Microalbuminuria is a risk marker for cardiovascular disease [9] and has been suggested to reflect a state of low-grade systemic inflammation [21]. Several factors like high blood pressure, hyperglycaemia, smoking, heart failure and renal atherosclerosis, all of which themselves are associated with increased inflammatory activity, are known to play a role in the development of microalbuminuria [22]. The association with microalbuminuria was, however, restricted to non-diabetic subjects. This could reflect the fact that in diabetic subjects other factors including hyperglycaemia may influence the day-to-day variation in albumin excretion. The non-diabetic patients with microalbuminuria had several features of MetS including higher waist to hip ratio, higher HOMA –index, and higher blood pressure (data not shown) compared to individuals without microalbuminuria. Accordingly, the *MHC2TA* -168A→G polymorphism was also associated with MetS.

Thus, the *MHC2TA* -168 A→G polymorphism influenced both outcome and prediction of cardiovascular disease. Among patients with previous MI, carriers of the AG or GG genotypes had increased risk of cardiovascular death compared with AA genotype carriers (Table 3 and Figure 1). It is known that inflammation plays a key role in development of atherosclerosis [11] and activated T-lymphocytes are already present in the atherosclerotic plaque, as well as in the immediate site of plaque rupture or superficial erosions in patients who have died due to MI or unstable angina [23]. Our results suggest that the G-allele (and in particular the AG genotype) could be a risk factor for cardiovascular mortality after MI, although the mechanism remains unclear. Swanberg *et al.* suggested that the G allele could cause reduced induction of MHC class II genes thus leading to less efficient presentation of antigens to regulatory T cells [13]. However, the previous association analysis compared AG and GG genotype carriers to AA genotype carriers and showed that in particular the AG (and not GG) genotype carriers were at higher risk [13]. In contrast, the expression analysis compared a pool of AA and AG genotype carriers to GG genotype carriers thus not challenging the possibility of a difference between the more common AA and AG genotypes [13]. Interestingly, we observed that in fact the GG carriers had a lower risk for death due to cardiovascular events compared to AA or AG genotype carriers (HR 0.38 [0.16–0.92]). It is therefore unclear whether the risk really is associated with less induction of the MHC II genes in response to inflammation stimuli. To clarify this issue, expression levels of all genotypes would therefore be of interest, especially comparison between the AA and AG genotypes.

Taken together, despite lack of a relationship between *MHC2TA* and MI in this large association study, we show that the AG/GG genotypes of the *MHC2TA* -168 A→G polymorphism are associated with microalbuminuria and features of MetS. This, in turn, translates into an increased risk of cardiovascular mortality.
